# KAI407, a Potent Non-8-Aminoquinoline Compound That Kills Plasmodium cynomolgi Early Dormant Liver Stage Parasites *In Vitro*

**DOI:** 10.1128/AAC.01927-13

**Published:** 2014-03

**Authors:** Anne-Marie Zeeman, Sandra M. van Amsterdam, Case W. McNamara, Annemarie Voorberg-van der Wel, Els J. Klooster, Alexander van den Berg, Edmond J. Remarque, David M. Plouffe, Geert-Jan van Gemert, Adrian Luty, Robert Sauerwein, Kerstin Gagaring, Rachel Borboa, Zhong Chen, Kelli Kuhen, Richard J. Glynne, Arnab K. Chatterjee, Advait Nagle, Jason Roland, Elizabeth A. Winzeler, Didier Leroy, Brice Campo, Thierry T. Diagana, Bryan K. S. Yeung, Alan W. Thomas, Clemens H. M. Kocken

**Affiliations:** a Biomedical Primate Research Centre, Department of Parasitology, Rijswijk, The Netherlands; b Genomics Institute of the Novartis Research Foundation, San Diego, California, USA; c Medical Parasitology-268, MMB-NCMLS, Radboud University Nijmegen Medical Centre, Nijmegen, The Netherlands; d Medicines for Malaria Venture, Geneva, Switzerland; e Novartis Institute for Tropical Diseases, Singapore

## Abstract

Preventing relapses of Plasmodium vivax malaria through a radical cure depends on use of the 8-aminoquinoline primaquine, which is associated with safety and compliance issues. For future malaria eradication strategies, new, safer radical curative compounds that efficiently kill dormant liver stages (hypnozoites) will be essential. A new compound with potential radical cure activity was identified using a low-throughput assay of *in vitro*-cultured hypnozoite forms of Plasmodium cynomolgi (an excellent and accessible model for Plasmodium vivax). In this assay, primary rhesus hepatocytes are infected with P. cynomolgi sporozoites, and exoerythrocytic development is monitored in the presence of compounds. Liver stage cultures are fixed after 6 days and stained with anti-Hsp70 antibodies, and the relative proportions of small (hypnozoite) and large (schizont) forms relative to the untreated controls are determined. This assay was used to screen a series of 18 known antimalarials and 14 new non-8-aminoquinolines (preselected for blood and/or liver stage activity) in three-point 10-fold dilutions (0.1, 1, and 10 μM final concentrations). A novel compound, designated KAI407 showed an activity profile similar to that of primaquine (PQ), efficiently killing the earliest stages of the parasites that become either primary hepatic schizonts or hypnozoites (50% inhibitory concentration [IC_50_] for hypnozoites, KAI407, 0.69 μM, and PQ, 0.84 μM; for developing liver stages, KAI407, 0.64 μM, and PQ, 0.37 μM). When given as causal prophylaxis, a single oral dose of 100 mg/kg of body weight prevented blood stage parasitemia in mice. From these results, we conclude that KAI407 may represent a new compound class for P. vivax malaria prophylaxis and potentially a radical cure.

## INTRODUCTION

Plasmodium vivax malaria causes between 70 million (1) and 390 million (2) clinical cases per year (for a review, see reference 3). Although *vivax* malaria is often referred to as “benign,” the disease is not mild, causing morbidity and mortality (4, 5). The parasite, which is dependent on the Duffy antigen for red blood cell (RBC) invasion, has been largely absent from West Africa, where the population is primarily Duffy negative. However, recent publications on P. vivax infections in Duffy-negative people (6–8) suggest that P. vivax is making its way into other areas of Africa, emphasizing the urgent need for new treatments.

One of the difficulties in fighting P. vivax stems from malaria relapses, caused by activation of dormant liver stage parasites, called hypnozoites (9). Currently, the only licensed drug for the radical cure of P. vivax malaria is primaquine (PQ), which is active against blood stage parasites (asexual and gametocyte stages) and liver stage parasites, including hypnozoites (10). However, PQ is contraindicated in glucose-6-phosphate dehydrogenase (G6PD)-deficient people, who can suffer from acute hemolytic anemia if treated with PQ (11). The CDC-recommended treatment schedule for PQ is 30 mg/day for 14 days (in non G6PD-deficient patients), limiting patient compliance, which could result in PQ resistance (12, 13).

Until now, the *in vivo* Plasmodium cynomolgi/rhesus monkey model has been the only model system exploited extensively to test new antimalarials for radical cure activity (14, 15, 53), as P. cynomolgi is one of the few parasite species that forms hypnozoites. Using this model, tafenoquine (16), elubaquine (also known as bulaquine or CDRI80/53) (17), and NPC1161 (18) were identified as antirelapse drugs, and these compounds are currently in preclinical development. However, all are 8-aminoquinolines, and all may have the same liabilities associated with PQ. Recently, Liu et al. (19) described a new non-8-aminoquinoline compound with radical cure properties in rhesus macaques, suggesting that new chemical structures may also act on hypnozoites.

The identification of new chemical entities with potential radical cure activity has been hampered by the lack of an *in vitro* assay to screen the parasite liver stages. We recently described the identification of imidazolopiperazines, which are active *in vitro* on both Plasmodium blood and liver stages (20). The *in vitro* liver stage assay used for screening is an image-based assay with HepG2-A16-CD81^EGFP^ cells infected with Plasmodium yoelii sporozoites. The assay can identify compounds that that arrest or eliminate liver schizonts at various stages in development. Some of these have subsequently been shown to be effective causal prophylaxis agents *in vivo*. Although P. yoelii does not form hypnozoites, we surmise that compounds with activity on blood stages and liver schizonts are more likely to act on the dormant liver stages than randomly selected compounds or compounds with blood stage activity only. We used this assay to preselect liver stage-active compounds for evaluation on P. cynomolgi liver stages. The assay described in this paper assesses compound activity on liver stage cultures *in vitro* using P. cynomolgi parasites and is similar to assays previously described (21–24, 52) but is adapted to be more efficient for *in vitro* drug screening by reducing cultivation time and utilizing a fully automatic readout. In this adapted P. cynomolgi assay, the development of two distinct populations of parasites is monitored in the cultures: small forms that resemble hypnozoites both in appearance and in drug sensitivity profile and developing liver stage schizonts (21). Recently, Dembele et al. (25) have shown that the small forms can reactivate *in vitro* and can grow out to mature liver schizonts, confirming the hypothesis that these small forms are hypnozoites.

We evaluated the reproducibility of the P. cynomolgi *in vitro* liver stage assay and used it in a low-throughput screen of compounds with demonstrated antimalarial activity, identifying a new potent non-8-aminoquinoline compound that, similar to primaquine, efficiently kills the early developmental forms of all liver stages *in vitro*.

## MATERIALS AND METHODS

Details of materials and methods are provided in the supplemental material.

### Generation of P. cynomolgi sporozoites.

The Biomedical Primate Research Centre (BPRC) is an AAALAC-certified institute. All rhesus macaques (Macaca mulatta) used in this study were captive bred for research purposes and were housed at the BPRC facilities in compliance with the Dutch law on animal experiments, European directive 86/609/EEC, and with the Standard for Humane Care and Use of Laboratory Animals by Foreign institutions, identification number A5539-01, provided by the National Institutes of Health (NIH). Prior to the start of experiments, all protocols were approved by the local independent ethical committee, according to Dutch law. Rhesus macaques were infected with 1 × 10^6^ P. cynomolgi M strain blood stage parasites and bled at peak parasitemia. Approximately 300 female Anopheles stephensi mosquitoes strain Sind-Kasur Nijmegen (Nijmegen University Medical Centre St. Radboud, Department of Medical Microbiology) were fed with this blood (26).

### Isolation and maintenance of primary rhesus hepatocytes.

Rhesus monkey hepatocytes were isolated from liver lobes, as described by Guguen-Guillouzo et al. (27). Sporozoite infections were performed within 3 days after hepatocyte isolation.

### Sporozoite infection of primary rhesus hepatocytes.

Sporozoite inoculation of primary rhesus hepatocytes was performed according to the methods of Mazier et al. (28) and Dembele et al. (21). Hepatocytes were washed with William's B medium before adding 50,000 (96-well plates) or 90,000 (LabTek chamber slides) sporozoites/well. Cultures were kept at 37°C in 5% CO_2_ with regular medium changes. To evaluate the development of P. cynomolgi liver stages, slides were fixed with cold methanol at the indicated time points.

### Drug assays on liver stage parasites.

Compounds were diluted in William's B medium to 10, 1, and 0.1 μM. Atovaquone, primaquine, and medium only were used as controls. For 50% inhibitory concentration (IC_50_) determination 2-fold serial dilutions (10 to 0.04 μM final concentrations) were evaluated in duplicate. Statistical analyses were performed as described in the supplemental material.

### Visualization of liver stages.

Intracellular P. cynomolgi malaria parasites were stained with the cross-reactive mouse polyclonal anti-Plasmodium falciparum Hsp70 antibody and secondarily stained with fluorescein isothiocyanate (FITC)-labeled goat anti-mouse IgG (21). Alternatively, anti-P. cynomolgi Hsp70.1 (anti-*Pcy*Hsp70) and FITC-labeled goat anti-rabbit Ig antibodies were used. Anti-P. cynomolgi Hsp70 antisera were generated against part of the Hsp70 gene (amino acids 350 to 6860) (see the supplemental material) (29). The number of exoerythrocytic forms (EEFs) was determined for each well, using a Leica DMI6000 inverted microscope. In later assays, the number of intracellular parasites was determined using a high-content imaging system (Operetta; Perkin-Elmer). Validation of the Operetta analysis was performed by comparing manual-counting results of a number of plates with the outcome of Operetta analysis. The parasitophorous vacuolar membrane (PVM) was visualized using rabbit anti-P. cynomolgi Etramp serum generated against a mixture of two peptides [(C)-IISPNDELKKEGLD and (C)-IMKHRKKERKEMED, where "C" is cysteine, for coupling to column].

### Criteria for distinguishing large and small EEF parasites.

Based on the limited information available about *in vivo* hypnozoite morphology, we used the following definitions (30, 31): hypnozoite, a small (maximum 7-μm-diameter), round intracellular parasite with 1 nucleus; developing parasite, an intracellular parasite, larger than 7 μm and round or irregular in shape, with more than 1 nucleus.

### Sporozoite infection and atovaquone treatment of rhesus macaques.

Sporozoites were isolated according to the method of Mazier et al. (28) and resuspended in phosphate-buffered saline (PBS); 6.37 × 10^6^ sporozoites were injected into the vena saphena of two rhesus macaques while under ketamine sedation. One of the animals was given atovaquone (Mepron; 150 mg/ml; 10 mg/kg of body weight) via gavage immediately after the sporozoite injection, followed by five daily oral dosings. The other animal was treated with chloroquine (7.5 mg/kg) when blood stage positive. Parasitemia was monitored via thin-film slides, which were fixed with methanol and stained with Giemsa stain.

### *In vivo* causal prophylaxis mouse model.

Female ICR mice (8 or 9 weeks old) were randomized into groups of four mice. The animals in each group were administered, via oral gavage, 20 or 100 mg/kg KAI407, 2.5 mg/kg atovaquone (in 0.5% [wt/vol] methylcellulose and 0.5% [vol/vol] Tween 80 in distilled water), or vehicle only. Within 2 h of oral dosing, all mice were inoculated intravenously via the lateral tail vein with 50 × 10^3^ Plasmodium berghei ANKA luciferase sporozoites (32) in 5% PBS. Blood smears were obtained on days 5 to 9, 12, 16, 23, and 30 postinoculation. Mice were observed twice daily for clinical signs of illness, morbidity, and mortality. Animals were humanely euthanized when blood parasitemia exceeded 2% or mice survived to day 30 (completion of the prophylaxis study).

## RESULTS

### P. cynomolgi-infected rhesus monkeys provide a reliable source for sporozoite production.

To overcome the bottleneck of accessing sufficient numbers of sporozoites from P. vivax for *in vitro* hypnozoite drug assays, we set up a P. cynomolgi sporozoite production platform. Due to the very reproducible blood stage infection pattern of rhesus monkeys (33), this proved to be a reliable system for infecting A. stephensi mosquitoes, yielding an average of 50,000 sporozoites per mosquito over 53 infections. A detailed overview of blood stage parasitemia, transmission, oocyst count, and sporozoite yield is given in Table S1 in the supplemental material. Parasitemia ranged between 0.02 and 3.6% on the first day of mosquito feeding (feed 1, usually 11 days after infection of a rhesus monkey), and all but four transmissions were successful. The oocyst count was positively correlated with blood stage parasitemia at the time of feed 1 (Spearman's rho, 0.732; *P* < 10^−8^) (see Fig. S1A in the supplemental material). Interestingly, the four lowest parasitemia counts were found in rhesus monkeys of Burmese origin, resulting in low oocyst counts, as well. Monkeys of Indian or Chinese origin yielded better results. The number of sporozoites was weakly positively correlated with oocyst counts from feed 1 (Spearman's rho, 0.398; *P* = 0.0062) (see Fig. S1B in the supplemental material). The positive correlation between parasitemia and oocyst number is also found for feed 2 (at day 12 after rhesus infection), albeit weaker (Spearman's rho, 0.37; *P* = 0.0092) (see Fig. S1C in the supplemental material). The correlation coefficient between sporozoite yield and oocyst number (see Fig. S1D in the supplemental material) for feed 2 is 0.51 (*P* = 0.0017). A subset of sporozoites from the 53 mosquito feeds was used for the assays described below.

### P. cynomolgi liver stage parasites develop to full maturity *in vitro*, with small liver stage parasites resembling hypnozoites remaining.

**FIG 1:**
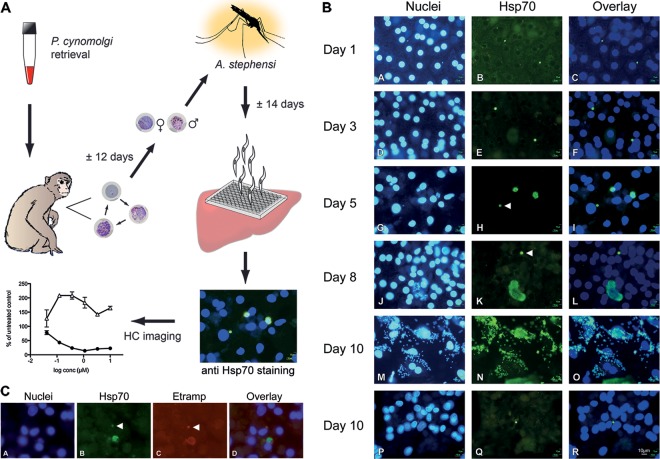
*In vitro* liver stage assay, intracellular development of P. cynomolgi, and PVM staining of small EEFs. (A) Schematic representation of the P. cynomolgi *in vitro* liver stage assay workflow. The experiment starts with the infection of a rhesus monkey with blood stage parasites from thawed stock. Mosquitoes are fed on the parasitized rhesus blood around peak parasitemia. Salivary gland sporozoites are harvested ∼14 days after the infected blood meal to infect primary rhesus hepatocytes. After sporozoite invasion, compounds are added in a concentration series, and the assay mixtures are incubated for 6 days. Parasites are visualized by staining Hsp70 with fluorescent antibodies. Small and large liver stage parasites are differentially counted using a high-content imager, resulting in a dose-response curve. (B) Sporozoite-infected primary rhesus hepatocytes were fixed 1, 3, 5, 8, or 10 days after inoculation with P. cynomolgi sporozoites. Nuclei were stained with 4′,6-diamidino-2-phenylindole (DAPI) (left column). Parasites were stained with anti-Hsp70 antibodies and fluorescein isothiocyanate (FITC)-labeled secondary antibodies (middle column). An overlay is shown in the right column. Within 24 h after infection, intracellular parasites are visible (images A to C), containing one nucleus (image A). The growth rates of all intracellular parasites appear to be similar until day 3 (images D to F). After 5 (images G to I) and 8 (images J to L) days of intracellular development, schizonts and hypnozoite forms (arrowhead) can be distinguished. Approximately 53% of the EEFs develop into mature schizonts, and 47% of the parasites remain small for the duration of the assay. Ten days after sporozoite inoculation, in the same well, free merozoites were observed (images M to O), as well as persistent small forms with a single nucleus (P to R). The developing liver stage parasites grow out to full maturity in a similar time frame, as reported *in vivo* (51). (C) Etramp staining of P. cynomolgi liver stage parasites. A liver schizont and a hypnozoite form (arrowheads) are seen in this field. Images: A, nuclei of P. cynomolgi-infected primary rhesus hepatocytes stained with DAPI; B, mouse anti-Hsp70/anti-mouse FITC staining of P. cynomolgi liver stage parasites; C, PVM stained with rabbit-anti-P. cynomolgi Etramp/anti-rabbit-tetramethyl rhodamine isocyanate (TRITC), showing the presence of a PVM in both parasites; D, merge of images A, B, and C. The presence of a PVM indicates that small EEFs are not the product of an aborted *in vitro* invasion but, rather, that the parasites have successfully invaded the hepatocyte.

Freshly isolated primary rhesus hepatocytes from different monkeys were used for *in vitro* sporozoite infections (Fig. 1A). Hepatocytes were incubated with freshly dissected sporozoites, resulting in variable numbers of EEFs. Infected-cell numbers ranged from 0.09 to 3.0% (observations from 16 successful assays) (see Fig. S2 in the supplemental material). No clear correlation could be established between the backgrounds of hepatocyte donors and hepatocyte quality, but cell quality (visual evaluation involved cell density, granulation, and shape) played a role in sporozoite permissiveness, as EEF counts were reduced in visibly unhealthy cells. To follow EEF development over time, P. cynomolgi sporozoite-infected hepatocytes were fixed at several time points after infection and stained with anti-HSP70 antibodies (Fig. 1B). From day 5 postinfection (p.i.), two populations of parasites began to emerge, consisting of small forms that remained at the size of ∼3-day-old parasites (<7 μm) and a population of heterogeneously sized schizonts. At day 10 p.i., we could observe small forms that had remained small (Fig. 1B and P to R), as well as free merozoites (Fig. 1B and M to O). Incubation of RBCs with merosomes from the *in vitro* liver stage cultures did not result in P. cynomolgi-infected RBCs, probably because of the parasite's preference for reticulocytes. The small, uninucleate, persistent EEFs are likely to be hypnozoites, as was also noted by Hollingdale et al. (23), as well as Dembele et al. (21), who showed differential drug sensitivity of these forms. Further proof that the small forms we observed are indeed hypnozoites was recently provided by Dembele et al. (25), who showed that the small forms can reactivate *in vitro*. The small EEFs we therefore call hypnozoite forms can be positively stained for P. cynomolgi Etramp, a protein known to localize to the parasitophorous vacuole membrane (PVM) of EEFs (34) (Fig. 1C). This indicates that small EEFs are not the product of an aborted *in vitro* invasion but have successfully invaded a hepatocyte and formed a PVM.

### A reproducible drug assay platform allows testing of antihypnozoite activity.

**FIG 2:**
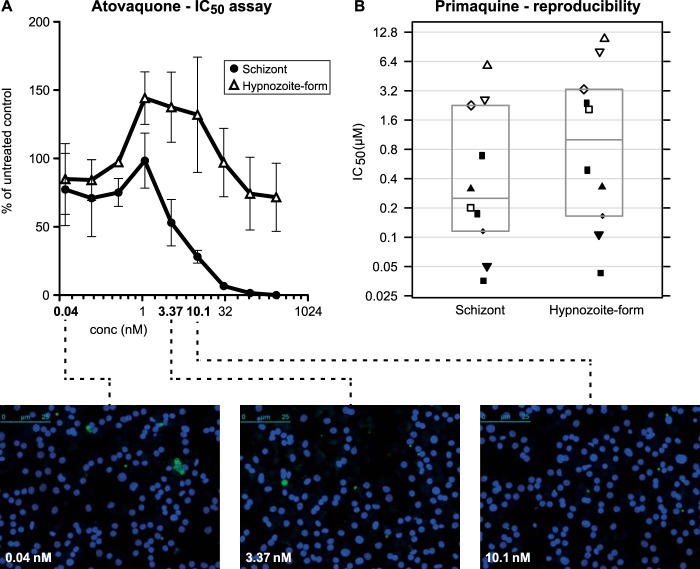
P. cynomolgi liver stage drug assay validation. (A) Atovaquone sensitivity profile of large EEFs and hypnozoite forms. Three independent atovaquone IC_50_ assays were averaged and are shown here (± standard deviations [SD]) with representative images of assay wells. Developing EEFs are more sensitive to the compound than hypnozoite forms. At higher atovaquone concentrations, only small parasites are visible. (B) Reproducibility of the primaquine IC_50_ assay. Ten independent P. cynomolgi primaquine *in vitro* liver stage drug assays were performed. IC_50_s were determined for individual assays and plotted. Indicated are the mean values and the 95% confidence intervals. IC_50_s from the same experiment for schizonts and hypnozoite forms are indicated with identical symbols. Note that the IC_50_ for hypnozoite forms is always higher than for schizonts within a single assay.

With a view to developing a higher-throughput drug assay than previously reported (21, 22), we amended the assay described by Dembele et al. (21) by exposing EEFs to compounds immediately after sporozoite invasion and incubating them until day 6, rather than from days 5 to 8. In addition, we implemented an automatic high-content imaging readout. To validate the new assay, we infected monolayers of primary rhesus hepatocytes with 50,000 P. cynomolgi sporozoites to which test compounds were added approximately 3 h after sporozoite inoculation. Six days after sporozoite infection, the plates were fixed and stained with anti-P. cynomolgi Hsp70 antibodies. Small and large EEFs were separately counted, based on size and number of parasite nuclei; 53% ± 12.7% (range, 28 to 74%) of the EEFs consisted of developing liver stage parasites, independent of the infection level of the cells, while 47% ± 12.7% (range, 26 to 72%) of the parasites remained small. Essentially, we observed drug sensitivity profiles identical to those with the day 5 to 8 treatment schedule described by Dembele et al. (21). More specifically, the small, uninucleate EEFs were found to be susceptible only to PQ and not to atovaquone (Fig. 2A) or pyrimethamine, similar to hypnozoites, and are different from developing EEFs, which are killed by all 3 drugs.

Collectively, these data show the presence of hypnozoite forms, both by appearance and in the drug sensitivity profile. Further support for the presence of hypnozoites in culture comes from comparison to an *in vivo* drug test in a P. cynomolgi sporozoite-infected rhesus monkey. The monkey was treated with atovaquone for 6 days, starting immediately after sporozoite injection, similar to the *in vitro* drug assay. While an untreated control monkey developed primary blood stage parasitemia on day 9, the atovaquone-treated monkey developed blood stage parasitemia only on day 19, at the time of the first relapse of the untreated animal. Thus, *in vitro*, only hypnozoite forms remain after atovaquone treatment, and *in vivo*, such parasites reactivate and cause malaria relapse. Recently, these observations were further substantiated by Dembele et al., who showed *in vitro* reactivation of hypnozoite forms in atovaquone-treated P. cynomolgi cultures (25).

To assess the robustness of the *in vitro* drug assay, we performed 10 independent PQ IC_50_ determinations. Assays were performed with independent hepatocyte and sporozoite batches using independent PQ dilutions. The mean IC_50_ for PQ was 0.374 μM (range, 0.036 to 5.780 μM) for developing liver stage parasites and 0.839 μM (range, 0.043 to 10.924 μM) for hypnozoite forms (Fig. 2B). We attributed the significant assay-to-assay variation of PQ IC_50_s to variability of the primary hepatocytes used in the assay. PQ is converted to an active metabolite and thus may be affected by hepatocyte viability and the presence of active cytochrome P450.

Evaluators blinded to reference compounds with known anti-blood stage and/or anti-liver stage activity evaluated them in a three-point, 10-fold-dilution fingerprinting series. The data are largely in agreement with what is known of their antimalarial activities (Table 1). Anti-blood stage compounds, like chloroquine and artesunate, did not show any significant anti-EEF activity. On the other hand, 8-aminoquinolines, known to prevent relapses *in vivo*, were all active against hypnozoite forms and schizonts (Table 1). They included PQ; NPC1161B (35); tafenoquine, currently in clinical trials (36); and bulaquine, also currently in clinical trials (37–41). Atovaquone was the only compound tested that specifically inhibited developing EEFs to a high degree without affecting hypnozoite forms. Cycloheximide, a protein biosynthesis inhibitor, showed dose-dependent inhibition of developing EEFs, as well as a limited effect on hypnozoite forms, but only at the highest dose tested. This suggests that low-level protein biosynthesis may be ongoing in the small, persistent liver forms. However, at the highest cycloheximide dose, hepatocyte viability may have been compromised due to its general toxicity, even though we did not observe significant hepatocyte loss based on nucleus counts. This may also have contributed to reduced numbers of hypnozoite forms. All reference drugs displayed the expected activity pattern on liver stages, suggesting a strong predictive value of this *in vitro* liver stage/hypnozoite form drug assay.

**TABLE 1:** Reference compounds evaluated in three-point 10-fold dilution series on P. cynomolgi liver stages

Compound name	Activity against^*a*^:	
	Hypnozoite forms	Schizonts
Amodiaquine^*b*^	−	−
Artesunate^*b*^	−	−
Dihydroartimisin^*b*^	−	−
Lumefantrine^*b*^	−	−
Piperaquine^*b*^	−	−
Chloroquine^*b*^	−	±
Doxycycline^*b*^	±	±
Mefloquine^*b*^	±	+
Proguanil^*b*^	±	+
Atovaquone^*c*^	−	+++
Cycloheximide^*d*^	+	++
Primaquine^*e*^	+++	+++
NPC-1161B^*f*^	+++	+++
Tafenoquine^*f*^	++	+++
Bulaquine^*f*^	+++	+++

a Activities of compounds (reduction of parasite counts at 10 μM) are represented as follows: −, 0 to 10%; ±, 10 to 20%; +, 20 to 70%; ++, 70 to 90%; +++, >90%. Of the nonaminoquinoline antimalarials, only atovaquone reduced the number of developing liver stage parasites by more than 90%. The nonspecific protein synthesis inhibitor cycloheximide had a minor effect on the number of small and large liver stage parasites at the highest concentration tested. All 8-aminoquinolines tested were active in the P. cynomolgi *in vitro* assay, efficiently killing both schizonts and hypnozoite forms.

b Reference compound had known blood stage activity.

c Reference compound had known blood and liver stage activity.

d Reference compound is a known protein synthesis inhibitor.

e Reference compound is an 8-aminoquinoline, used as a radical cure, active against gametocytes and liver stages (including hypnozoites).

f Reference compound is an 8-aminoquinoline.

### Small-scale screening of malaria box compounds (preselected compounds with bloodstage activity) identified a new potent anti-liver stage compound class.

We screened 14 selected compounds that previously showed activity against the malaria blood and/or liver stage (42). These compounds were primary hits or had been subject to some preliminary lead optimization. The selection was based on diversity of chemical structure and opportunities to further modify the compounds. Compounds were initially screened in three-point 10-fold dilutions (see Table S2 in the supplemental material). Interestingly, one of the compounds active against developing liver stages, QN254, is a known dihydrofolate reductase (DHFR) inhibitor (11). The fact that it was not active against hypnozoite forms highlights the assay's expected ability to distinguish compounds that are active only against large EEFs.

**FIG 3:**
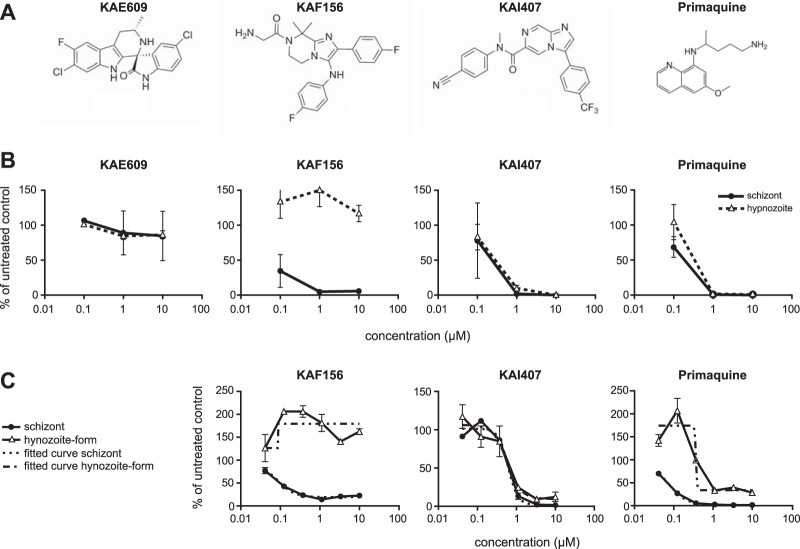
P. cynomolgi drug assays with selected malaria box compounds. (A) Structure formulas of KAE609, KAF156, KAI407, and primaquine. (B) Three-point 10-fold dilution series (0.1, 1, and 10 μM) on P. cynomolgi liver stage cultures. The percentage of untreated control is shown as a function of the compound concentration. Differential counting of schizonts and hypnozoite forms was performed based on size and number of parasite nuclei. The results of one representative assay are shown; assays were performed at least twice. KAE609 was not active against liver stage parasites and was therefore not taken forward for IC_50_ determination. (C) IC_50_ determination for KAF156, KAI407, and primaquine. Drugs were tested in a 2-fold dilution series. The percentage of untreated control is shown as a function of the compound concentration. Differential counting of schizonts and hypnozoite forms was performed based on size and number of parasite nuclei. IC_50_s were calculated based on fitted curves (nonlinear fit). The results of one representative assay are shown, and at least two independent assays were performed. The activity of KAF156 is limited to schizonts, while KAI407 and primaquine also kill hypnozoite forms. The error bars indicate standard deviations.

We focused on three different compounds, evaluating their activities against P. cynomolgi hypnozoite forms. These compounds were the spiroindolone KAE609, which displays subnanomolar activity against P. falciparum and P. vivax blood stages (43); the imidazolopiperazine KAF156, an optimized clinical candidate that has low nanomolar activity against blood and liver stages (44); and the imidazopyrazine KAI407 (Fig. 3A). KAI407 was derived from a lead compound (imidazolopyridine, compound 1 in Table 2) identified in a high-throughput blood stage screen (42), which also showed weak activity in an *in vitro* assay using P. yoelii sporozoites and HepG2 cells (20). Introduction of a methyl group to the amide nitrogen produced compound 2, which improved blood stage activity nearly 100-fold but lost liver stage activity. By modification of both aryl appendages, we obtained compound 3**,** with blood and liver stage activity. Chemical modifications of the 6,5-heterocyclic core provided an activity breakthrough. Introducing a nitrogen to the 5 position of the bicyclic core generated the imidazopyrazine KAI407 (compound 4), with good potency (IC_50_ < 100 nM) on both blood and liver stage parasites (Table 2).

**TABLE 2:**
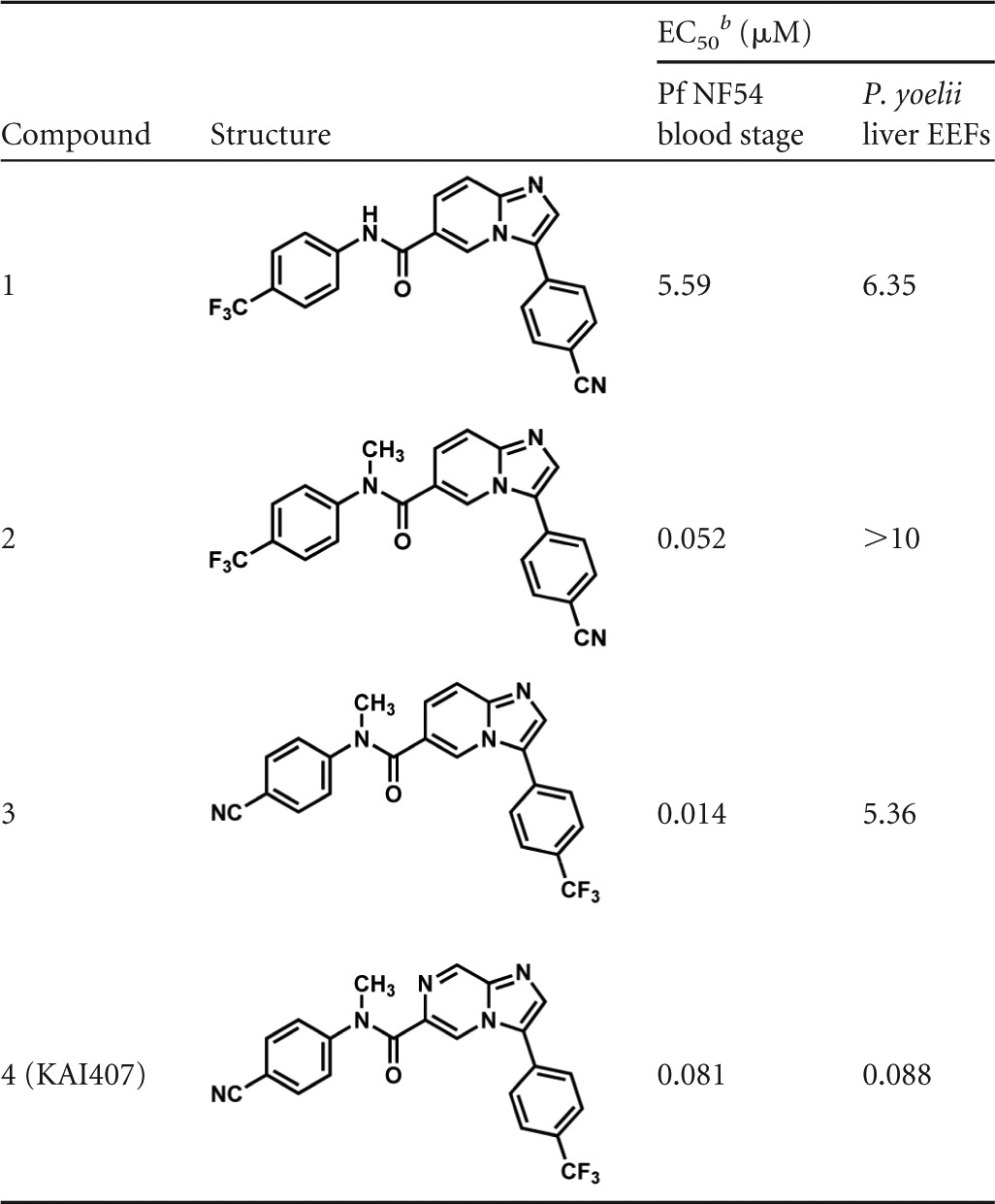
Lead optimization toward KAI407^*a*^

a KAI407 was derived from lead compound 1, identified in a high-throughput blood stage screen, which also showed weak activity in an *in vitro* assay using P. yoelii sporozoites and HepG2 cells. Introduction of a methyl group to the amide nitrogen provided compound 2 and improved blood stage activity nearly 100-fold, but lost liver stage activity. By modification of both aryl appendages, we obtained compound 3**,** with blood and liver stage activity. Chemical modifications of the 6,5-heterocyclic core by introducing a nitrogen to the 5 position of the bicyclic core generated the imidazolopyrazine KAI407 (compound 4) with good potency (IC_50_ < 100 nM) on both blood and liver stage parasites.

b EC_50_, 50% effective concentration.

To determine if liver stage activity on the rodent parasite would predict activity against P. cynomolgi liver stages, a three-point 10-fold dilution series was performed with KAE609, KAF156, and KAI407 (Fig. 3B). Similar to what was previously reported for rodent malaria liver stages (20), KAE609 showed no activity against either P. cynomolgi developing liver stages or hypnozoite forms. KAF156 was active only against developing liver stages, in agreement with the known causal prophylactic activity of members of these series, such as GNF179 (16). In contrast, KAI407 displayed activity against both P. cynomolgi developing liver stages and hypnozoite forms, similar to PQ. Subsequent IC_50_ determinations for the two liver stage-active compounds showed IC_50_s of 0.09 μM for KAF156 and 0.64 μM (0.86 to 0.42 μM) for KAI407 on developing liver stages and >10 μM (no dose response) for KAF156 and 0.69 μM (0.84 to 0.55 μM) for KAI407 on hypnozoite forms (80 nM against P. yoelii *in vitro* EEFs). This is slightly better than PQ, for which IC_50_s in the same assays were 1.3 μM (0.29 to 2.31 μM) for developing liver stages and 1.8 μM (3.27 to 0.33 μM) for hypnozoite forms (Fig. 3C) (7). Overall, based on activity testing of this limited series, there seems to be concordance between the rodent malaria and P. cynomolgi liver stage activities, although activity on hypnozoite forms cannot be predicted.

### *In vivo* causal prophylaxis mouse model.

KAI407 as a lead compound needs further optimization before *in vivo* efficacy evaluation can be done in the P. cynomolgi rhesus relapse model. We thus could not yet evaluate the prophylactic and radical curative activities of this compound in the rhesus model. Based on its ability to eliminate developing liver stages, analogous to atovaquone and KAF156, we therefore further tested whether KAI407 could prevent the establishment of blood stage parasitemia after sporozoite inoculation in a causal prophylactic rodent model. Four groups of four mice each were treated by oral gavage with 20 or 100 mg/kg KAI407, 2.5 mg/kg atovaquone, or vehicle only and then infected with P. berghei ANKA luciferase sporozoites (32) within 2 h of dosing. The vehicle-only group became blood stage positive 5.75 ± 0.46 days p.i. The low-dose KAI407-treated group (20 mg/kg) became blood stage positive at day 6.5 ± 0.53 p.i., and neither the atovaquone-treated mice nor the 100-mg/kg KAI407-treated mice became blood stage positive over 30 days of monitoring, achieving 100% protection in the two groups (Table 3). Although these data do not speak to its potential radical cure activity, they demonstrate that KAI407 is active against malaria tissue stages *in vivo*.

**TABLE 3:** Causal prophylaxis activity of KAI407 in an *in vivo* rodent malaria model using P. berghei

Compound	Dose^*a*^ (mg/kg)	Survival (%)	Prepatent period^*b*^ (days ± SD)
Vehicle (untreated)		0	5.75 ± 0.46
Atovaquone	2.5	100	NA^*d*^
KAI407^*c*^	20	0	6.5 ± 0.53
KAI407^*c*^	100	100	NA^*d*^

a A single dose was administered via oral gavage 1–2 h prior to P. berghei sporozoite infection.

b Average number of days before blood-stage parasitemia was detected by microscopy.

c Formulated in 0.5% (wt/vol) methylcellulose, 0.1% (vol/vol) Tween 80.

d NA, not applicable; 100% prophylaxis achieved.

## DISCUSSION

The quiescent nature of malaria hypnozoites in the livers of P. vivax- or Plasmodium ovale-infected individuals represents a significant challenge for malaria eradication. Hypnozoites are found only in these two human malaria parasites and in a few nonhuman primate malaria parasites, one of which is P. cynomolgi. Primaquine remains the only treatment option for P. vivax patients but has potentially severe side effects and contraindications (for a recent review, see reference 45). Bolstering the malaria pipeline with new chemical entities that overcome the liabilities associated with primaquine is now a top priority. Historically, P. cynomolgi-infected monkeys were used for screening compounds to detect hypnozoitocidal activity. A large screening effort (14) in which hundreds of monkeys were treated with experimental compounds only confirmed the potency of 8-aminoquinolines as radical cure compounds. More recently, a compound from a different chemical class, a 2-guanidino-4-oxoimidazoline derivative (19), was found to cure relapsing P. cynomolgi infections in rhesus monkeys. *In vivo* large-scale screens are currently ethically and financially restricted. Moreover, they are extremely low throughput and time-consuming, which explains the dearth of new chemical entities with proven hypnozoitocidal activity. The development and application of an *in vitro* assay that can assess activity against hypnozoite forms can bridge the current gap between blood stage (P. falciparum) and liver schizont (P. yoelii) active compounds and the P. cynomolgi-infected rhesus monkey model. We chose P. cynomolgi rather than P. vivax as a way to reliably access sufficient numbers of sporozoites from a single parasite strain, which is currently not possible for P. vivax. Our data show that mosquito feeding on P. cynomolgi-infected rhesus monkey blood provides a reliable source of sufficient numbers of sporozoites for *in vitro* assays.

Time course immunofluorescence assay images of P. cynomolgi-infected hepatocytes show uniform EEF development (based on size and shape) until day 3 and two EEF populations emerging after that, with ∼53% of schizonts progressing to merozoites by day 10 and ∼47% of EEFs remaining as small uninucleate forms, essentially as previously described for *in vivo*-derived EEFs (46). The persisting small EEFs share characteristics of hypnozoites, including the morphologies derived from the limited description of *in vivo*-derived hypnozoites (30, 31) and their atovaquone and primaquine drug sensitivity profiles (21). Further evidence that these *in vitro*-cultured small forms could indeed be hypnozoites comes from our comparative *in vivo* drug treatment experiment, in which we observed no primary blood stages in an atovaquone-treated animal, which relapsed simultaneously with an untreated animal. *In vitro*, liver schizonts were completely absent and only small hypnozoite forms remained after atovaquone treatment. Dembele (25) showed in long-term *in vitro* cultures that these hypnozoite forms spontaneously started to develop into liver stage schizonts after the atovaquone treatment was stopped. Collectively, these data link the *in vitro*-cultured hypnozoite forms to the appearance of relapse *in vivo* following initial atovaquone treatment of infection and thus strengthen the case for *in vitro*-cultured hypnozoites. Additionally, despite the small sample size of *in vivo* prophylactic atovaquone-treated animals, the outcome corroborates occasional clinical observations of P. vivax and *P. ovale* infections in travelers, despite atovaquone/proguanil prophylaxis (47). When taken together with our *in vitro* data, this suggests that atovaquone is effective against the early developing liver stage forms destined to become primary hepatic schizonts but ineffective against the forms destined to become latent hypnozoites. Thus, when prophylaxis is discontinued, relapses are likely to occur.

Adaptations to the original *in vitro* model (21) were necessary in order to allow larger-scale *in vitro* compound screening. The implemented adaptations were (i) culture times reduced from 8 to 6 days to increase throughput; (ii) exposure to compounds continuously from day 0 to day 6 rather than from days 5 to 8 (here, we found that shorter exposure to primaquine reduced assay sensitivity, so we did not decrease the assay time any further); (iii) replacement of manual readout by automated high-content imaging, which dramatically increased assay throughput. We used the adapted assay to screen (blinded) a number of reference compounds and identified a number of active compounds, which were all 8-aminoquinolines known to prevent *in vivo* relapses, suggesting good predictive value of the assay for activity against day 0 to 6 hypnozoites.

In an initial (blinded) small-scale screen of selected antimalarial compounds (some of which had already been subjected to lead optimization), we confirmed the reported activities on rodent malaria liver stages in P. cynomolgi liver stages: the spiroindolone KAE609 showed no activity against P. cynomolgi liver stages, while the imidazolopiperazine KAF156 was active against P. cynomolgi liver stages (20, 48). Like atovaquone, KAF156 affected only developing liver stages in a dose-dependent manner and had no activity on hypnozoite forms.

With the modified drug treatment schedule, starting immediately after invasion, it became possible to identify different drug sensitivities of the liver stage parasites. We believe this difference reflects commitment of the sporozoites to become either a hypnozoite or a proliferative liver stage schizont at a very early time point. The very early hypnozoite is relatively insensitive to all drugs tested, showing reduced sensitivity to primaquine in comparison to developing parasites. This includes resistance to atovaquone, a licensed causal prophylaxis against P. falciparum (49). In contrast, the developing parasite population is sensitive, at similar concentrations, to anti-liver stage antimalarials (atovaquone and KAF156) that block P. yoelii from growing in hepatocytes. This suggests that commitment to the hypnozoite developmental pathway occurs early on, before, during, or immediately after infection of the hepatocyte. Despite this early commitment, parasites destined for the two pathways have indistinguishable rates of growth up to day 3. After 3 days of development, clear morphological differentiation of hypnozoites and developing liver stage parasites becomes evident. Thus, parasites committed to a period of dormancy require a period of growth subsequent to invasion before entering latency.

Whether, immediately subsequent to day 3, parasites have already entered a dormant state that can be reactivated is as yet unclear. In our hands and with our P. cynomolgi strain, the earliest relapse observed *in vivo* (in an animal that was treated with atovaquone from days 0 to 6 p.i. to kill all the primary liver stages) with this P. cynomolgi strain occurred 19 days p.i. In addition, another animal treated with chloroquine from days 14 to 16 to cure the primary blood stage parasitemia relapsed by day 21 p.i. Chloroquine treatment may have delayed the appearance of blood stage parasites in this animal. Assuming that (i) blood stage parasites detected at day 19 are caused by merozoites directly emerging from the liver (a reasonable assumption, because the animals were infected with over 6 million sporozoites, leading to a large liver load) and (ii) reactivated hypnozoites need 6 further days of development before merozoites are released, reactivation of hypnozoites *in vivo* could start around day 13 p.i. This suggests the possibility that, for a period between days 3 and 13 post-hepatocyte infection, further hypnozoite development may be required until a reactivation-competent parasite is established. Under our current assay format, both of the early stages (days 0 to 3 of treatment) that are committed to hypnozoite development but are indistinguishable from nonlatent forms and those that have engaged in a morphologically distinguishable hypnozoite-specific developmental pathway (days 4 to 6 of treatment) are analyzed for drug sensitivity. However, further research is needed to better understand hypnozoite development and to determine whether there is a period of development during which the hypnozoite-committed lineage cannot be activated.

In a focused hit-to-lead effort, we identified a series of blood stage-active compounds that were optimized for activity against rodent liver stages. For one compound, KAI407, the liver stage activity against rodent parasites translated into activity on both P. cynomolgi developing EEFs and hypnozoite forms. This compound displayed an activity profile identical to that of primaquine in the same assay. Interestingly KAI407 activity was less variable between assays than primaquine, suggesting a different metabolic pathway or mode of action, as conversion to an active metabolite does not seem to be required for activity (KAI407 is stable in the presence of liver microsomes). The 100% protection of mice by a single oral dose of 100 mg/kg KAI407 indicates that the compound is active as causal prophylaxis against P. berghei,and is an interesting candidate for causal prophylaxis against P. vivax-type parasites.

The exciting potential of the *in vitro* P. cynomolgi liver stage drug assay, as well as the newly discovered compound, is clear. Like primaquine, the newly discovered compound eliminated the early hepatic stages destined to become the latent hypnozoites. However, further optimization of the P. cynomolgi liver stage assay and miniaturization to increase the throughput are required before screens of larger, more chemically diverse libraries can be achieved. Improved hypnozoite cultures will permit more detailed study of hypnozoite biology, including work to identify reactivation triggers (50). More detailed investigation of the antirelapse properties of the KAI407 series of compounds as a critical step to determining their clinical potential will require assessment *in vivo*. KAI407 is currently the focus of a lead optimization effort to provide compounds suitable for testing in P. cynomolgi-infected rhesus monkeys for prophylactic and antirelapse activities, the gold standard for identifying antirelapse compounds. The outcome of such an *in vivo* experiment will be an important next step in the further validation of the *in vitro* P. cynomolgi liver stage drug assay and the discovery and further development of new antirelapse drugs for clinical use.

## Supplementary Material

Supplemental material
