# Supplementary material for: KAI407, a Potent Non-8-Aminoquinoline Compound That Kills Plasmodium cynomolgi Early Dormant Liver Stage Parasites In Vitro
Source: Antimicrob Agents Chemother. 2014 Mar;58(3):1586–95. doi: 10.1128/AAC.01927-13 (PMC3957848; doi:10.1128/AAC.01927-13)
Supplement: Supplemental material [file supp_58_3_1586__index.html]

KAI407, a Potent Non-8-Aminoquinoline Compound That Kills Plasmodium cynomolgi Early Dormant Liver Stage Parasites In Vitro — Supplemental material 

# KAI407, a Potent Non-8-Aminoquinoline Compound That Kills Plasmodium cynomolgi Early Dormant Liver Stage Parasites *In Vitro*

## Supplemental material

**Files in this Data Supplement:**

- Supplemental file 1 -

  Additional experimental details, Supplemental Figures S1 and S2, and Tables S1 and S2.

  PDF, 371K
