# Supplementary material for: KAI407, a Potent Non-8-Aminoquinoline Compound That Kills Plasmodium cynomolgi Early Dormant Liver Stage Parasites In Vitro
Source: Antimicrob Agents Chemother. 2014 Mar;58(3):1586–95. doi: 10.1128/AAC.01927-13 (PMC3957848; doi:10.1128/AAC.01927-13)
Supplement: Supplemental material [file AAC.01927-13_zac003142657so1.pdf]

## Supporting information to: KAI407, a potent non 8-aminoquinoline compound that kills *Plasmodium cynomolgi* dormant liver stage parasites *in vitro*

Zeeman *et al.*

**Supporting information:**

**Identification of *P. cynomolgi* Hsp70 and generation of anti-PcyHsp70 antibodies.**
**Protocols**
**Operetta data acquisition and analysis**
**Figure S1:** Correlation plots of blood stage parasiaemia in monkeys vs oocyst number in mosquitoes and oocyst number vs sporozoite yield.
**Figure S2:** Ratio of hypnozoite-forms/developing EEF vs total number of liver stage parasites over 16 independent assays.
**Table S1:** Transmission parameters of 53 *P. cynomolgi* infections in rhesus macaques.
**Table S2:** Selected Malaria box compounds evaluated in tenfold dilution series on *P. cynomolgi* liver stages.

### Identification of *P. cynomolgi* Hsp70 and generation of anti-PcyHsp70 antibodies.

Based on the *P. yoelii* Hsp70 (PY06158), a search was performed in the preliminary *P. cynomolgi* genome. Alignment of the putative *P. cynomolgi* Hsp70 protein (PcyHSP70) with *P. falciparum* 3D7 Hsp70 (PfHSP70_3D7) and *P. yoelii* Hps70 (PyHSP70) is shown below. The amino acid sequence used for overexpression in *E. coli* (using a codon optimized synthetic gene) and subsequent immunization (of rabbits and mice) is indicated in bold. Upstream of the polypeptide used for overexpression, a his-tag and a thrombin tag were inserted.

```
ClustalW (v1.83) multiple sequence alignment

3 Sequences Aligned           Alignment Score = nan
Gaps Inserted = 4             Conserved Identities = 651

Pairwise Alignment Mode: Slow
Pairwise Alignment Parameters:
    Open Gap Penalty = 10.0   Extend Gap Penalty = 0.1
    Similarity Matrix: gonnet

Multiple Alignment Parameters:
    Open Gap Penalty = 10.0   Extend Gap Penalty = 0.1
    Delay Divergent = 40%     Gap Distance = 8
    Similarity Matrix: gonnet



PfHSP70_3D7    1 MASAKGSKPNLPESNIAIGIDLGTTYSCVGVWRNENVDIIANDQGNRTTPSYVAFTDTER  60
PyHSP70        1 MANAKASKPNLPESNIAIGIDLGTTYSCVGVWRNENVDIIANDQGNRTTPSYVAFTDTER  60
PcyHSP70       1 MASGKASKPNLPESNIAIGIDLGTTYSCVGVWRNENVDIIANDQGNRTTPSYVAFTDTER  60
                 **  * ******************************************************

PfHSP70_3D7   61 LIGDAAKNQVARNPENTVFDAKRLIGRKFTESSVQSDMKHWPFTVKSGVDEKPMIEVTYQ 120
PyHSP70       61 LIGDAAKNQVARNPENTVFDAKRLIGRKFTESSVQSDMKHWPFTVKSGIEEKPMIEVVYQ 120
PcyHSP70      61 LIGDAAKNQVARNPENTVFDAKRLIGRKFTESSVQSDMKHWPFTVKSGVDEKPMIEVSYQ 120
                 ************************************************..******* **

PfHSP70_3D7  121 GEKKLFHPEEISSMVLQKMKENAEAFLGKSIKNAVITVPAYFNDSQRQATKDAGTIAGLN 180
PyHSP70      121 GEKKLFHPEEISSMVLQKMKENAEAFLGKSIKNAVITVPAYFNDSQRQATKDAGTIAGLN 180
```

```
PcyHSP70      121 GEKKLFHPEEISSMVLQKMKENAEAFLGKSIKNAVITVPAYFNDSQRQATKDAGTIAGLN 180
                  ************************************************************

PfHSP70_3D7   181 VMRIINEPTAAAIAYGLHKKGKGEKNILIFDLGGGTFDVSLLTIEDGIFEVKATAGDTHL 240
PyHSP70       181 VMRIINEPTAAAIAYGLHKKGKGEKNILIFDLGGGTFDVSLLTIEDGIFEVKATAGDTHL 240
PcyHSP70      181 VMRIINEPTAAAIAYGLHKKGKGEKNILIFDLGGGTFDVSLLTIEDGIFEVKATAGDTHL 240
                  ************************************************************

PfHSP70_3D7   241 GGEDFDNRLVNFCVEDFKRKNRGKDLSKNSRALRRLRTQCERAKRTLSSSTQATIEIDSL 300
PyHSP70       241 GGEDFDNRLVNFCVEDFKRKNRGKDLSKNSRALRRLRTQCERAKRTLSSSTQATIEIDSL 300
PcyHSP70      241 GGEDFDNRLVNFCVEDFKRKNRGKDLSKNSRALRRLRTQCERAKRTLSSSTQATIEIDSL 300
                  ************************************************************

PfHSP70_3D7   301 FEGIDYSVTVSRARFEELCIDYFRDTLIPVEKVLKDAMMDKKSVHEVVLVGGSTRIPKIQ 360
PyHSP70       301 FEGIDYSVTVSRARFEELCIDYFRDTLIPVEKVLKDAMMDKKSVHEVVLVGGSTRIPKIQ 360
PcyHSP70      301 FEGIDYSVTVSRARFEELCIDYFRDTLIPVEKVLKDAMMDKKSVHEVVLVGGSTRIPKIQ 360
                  ************************************************************

PfHSP70_3D7   361 TLIKEFFNGKEACRSINPDEAVAYGAAVQAAILSGDQSNAVQDLLLLDVCSLSLGLETAG 420
PyHSP70       361 TLIKEFFNGKEACRSINPDEAVAYGAAVQAAILSGDQSNAVQDLLLLDVCSLSLGLETAG 420
PcyHSP70      361 TLIKEFFNGKEACRSINPDEAVAYGAAVQAAILSGDQSNAVQDLLLLDVCSLSLGLETAG 420
                  ************************************************************

PfHSP70_3D7   421 GVMTKLIERNTTIPAKKSQIFTTYADNQPGVLIQVYEGERALTKDNNLLGKFHLDGIPPA 480
PyHSP70       421 GVMTKLIERNTTIPAKKSQIFTTYADNQPGVLIQVYEGERALTKDNNLLGKFHLDGIPPA 480
PcyHSP70      421 GVMTKLIERNTTIPAKKSQIFTTYADNQPGVLIQVYEGERALTKDNNLLGKFHLDGIPPA 480
                  ************************************************************

PfHSP70_3D7   481 PRKVPQIEVTFDIDANGILNVTAVEKSTGKQNHITITNDKGRLSQDEIDRMVNDAEKYKA 540
PyHSP70       481 PRKVPQIEVTFDIDANGILNVTAVEKSTGKQNHITITNDKGRLSPEEIDRMVNDAEKYKA 540
PcyHSP70      481 PRKVPQIEVTFDIDANGILNVTAVEKSTGKQNHITITNDKGRLSPEEIDRMVNDAEKYKA 540
                  ******************************************** .**************

PfHSP70_3D7   541 EDEENRKRIEARNSLENYCYGVKSSLEDQKIKEKLQPAEIETCMKTITTILEWLEKNQLA 600
PyHSP70       541 EDEENKKRIEARNSLENYCYGVKSSLEDQKIKEKLQPNEVETCMKSVTSILEWLEKNQLA 600
PcyHSP70      541 EDEENKKRIEARNSLENYCYGVKSSLEDQKIKEKLQPSEIETCMKSITTILEWLEKNQLA 600
                  *****.******************************* *.*****..*.***********

PfHSP70_3D7   601 GKDEYEAKQKEAESVCAPIMSKIYQDAAGAAGGMPGGMPGGMPGGMPG---------GMN 651
PyHSP70       601 GKDEYEAKQKEAEAVCSPIMSKIYQDAGAAAGGMPGGMPGGMPGGMPG-----GMPGGMN 655
PcyHSP70      601 SKEEYESKQKEAESVCAPIMSKIYQDVGGAAGGMPGGMPGGMPGGMPGGMPGGGMPGGMN 660
                   *.***.******.**.*********   ******************          ***

PfHSP70_3D7   652 FPGGMPG-AGMPGNAPAGSGPTVEEVD 677
PyHSP70       656 FPGGMPGGMGAPAGAPAGSGPTVEEVD 682
PcyHSP70      661 FPGGMPG-GGMPGGAPAGSGPTVEEVD 686
         ******* * *.************
```

## Protocols

**Generation of *P. cynomolgi* sporozoites.** A rhesus macaque was infected with $10^6$ *P. cynomolgi M* strain blood stage parasites, thawed from a first passage stock (i.e. blood stage parasites from a monkey infected with *P. cynomolgi* M strain sporozoites). At peak parasitemia (usually days 11 and 12 post infection) 9 ml infected blood was collected into a (Lithium-)heparinised tube. The blood was kept at 38°C and fed approximately 300 three to five days old female *Anopheles stephensi* mosquitoes Sind-Kasur strain Nijmegen (Nijmegen University Medical Centre St Radboud, Department of Medical Microbiology) using a glass feeder system (1). After the second bleed, monkeys were cured by three daily intramuscular injections of 7.5 mg/kg chloroquine (2), monitored for parasite clearance 24 days after chloroquine treatment and returned to colony.

Mosquitoes were kept in climate chambers at 25°C and 80% humidity and were fed 5% D-glucose on cotton pads daily. Six or seven days after feeding on the infected rhesus blood, oocyst numbers were determined in midguts of

ten individuals, stained in 1% mercurochrome in water. Mosquitoes were given a second (uninfected) blood meal to promote sporozoite invasion of the salivary glands. Salivary gland sporozoites were isolated from 12 days post feeding onwards.

**Isolation and maintenance of primary rhesus hepatocytes.** Rhesus monkey hepatocytes were isolated from liver lobes as follows. Immediately after resection, the liver was flushed with Viaspan (Bristol-Meyers-Squibb) to maintain hepatocyte viability. Livers were processed within two hours from resection by the two-step collagenase protocol, as described by Guguen-Guillouzo *et al*. (3). After collagenase VIII (C2139, Sigma) digestion of hepatocytes, dead and non-hepatocyte cells were removed using 36% percoll gradient in DMEM. Cells were washed in William's E containing 10% fetal calf serum (FCS) before resuspending in William's B medium (William's E with glutamax, containing 10% FCS, 2% penicillin/streptomycin (P/S), 1% insulin/transferrin/selenium, 1% NaPyruvate, 1% MEM-NEAA, 50 μM β-mercapto-ethanol, $10^{-7}$M dexamethasone (Alfasan, Woerden, The Netherlands)).

Cells were seeded at 290,000 cells/cm$^2$ into collagen coated (5μg/cm$^2$ rat tail collagen I) 96-well Greiner cellstar plates (90.000 cells/well) or 8-well Labtek chamber slides (210.000 cells/well) in William's B medium and left to attach for at least 2 h in a 37°C incubator at 5% $CO_2$. When attached, the medium was replaced by William's B with 2% dimethylsulfoxide (DMSO) to prevent hepatocyte dedifferentiation. Sporozoite infections were performed within three days after hepatocyte isolation.

**Sporozoite inoculation of primary rhesus hepatocytes.** Sporozoite inoculation of primary rhesus hepatocytes was performed according to Mazier *et al.* (4) between 14 and 28 days after feeding the mosquitoes on *P. cynomolgi* infected blood. Mosquitoes were transferred into beakers covered with mosquito nets, soaked in 70% ethanol and washed 4 times in Leibovitz' L15 medium containing 3% FCS and 2% P/S. Salivary glands were dissected under a Leica EZ4 stereo microscope, collected in the above medium in sterile 2 mL glass potter tubes on ice and ruptured by pottering 10 times. The suspension was passed over a 40 μm filter and spun for 3 min at 13,000 rpm in an Eppendorf centrifuge to collect sporozoites, which were washed and resuspended in William's B medium and counted in a Bürker-Turk counter chamber after settling in a humidified box for at least 5 min. Hepatocytes were washed with William's B prior to sporozoite inoculation, which were added at 50,000 sporozoites/well for 96-well plates or at 90,000 sporozoites/well for LabTek chamber slides. 96-well plates were spun 10 min at 500xg and placed into a 37°C, 5% $CO_2$ incubator for at least three hours before the first medium refreshment. LabTek chamber slides were left at room temperature for 2 h to settle the sporozoites, and placed into a 37°C, 5% $CO_2$ incubator overnight. Medium was refreshed every other day. To evaluate the development of *P. cynomolgi* liver stages, slides were fixed by cold methanol fixation at indicated time points.

**Drug assays on liver stage parasites.** Compound stocks (10 or 20 mM stocks in DMSO) were aliquoted and stored at -20°C. Compounds were diluted in William's B medium, passed over a 0.2 μm filter and further diluted to 10, 1 and 0.1 μM. Atovaquone, primaquine and medium only were used as controls. For $IC_{50}$ determination two-fold serial dilutions (10-0.04 μM final

concentration) were evaluated in duplicate. Test compounds were added to the EEF cultures at the time of first medium exchange. All medium refreshments contained the appropriate compound dilutions. Liver stage parasite numbers were determined as described below.

**Visualisation of liver stages.** Intracellular *P. cynomolgi* malaria parasites were stained with the cross-reactive mouse polyclonal anti-*P. falciparum* Hsp70.1 antibodies 1:2,000 in PBS (25). FITC labeled Goat-anti-mouse IgG was used as secondary antibody, 1:200 in PBS containing 2 μM 4',6-diamidino-2-phenylindole (DAPI) for nuclear staining. Alternatively, anti-*P. cynomolgi* Hsp70.1 (anti-*Pcy*Hsp70) antibodies were used. These were generated by overexpression of part of the Hsp70 gene (amino acid 350-686, see supplementary data) in analogy to previous papers (5). Briefly, the *P. cynomolgi* Hsp70.1 gene was identified from the preliminary *P. cynomolgi* genome, by homology search with *P. yoelii* Hsp70. An *E. coli* codon optimized synthetic gene containing a N-terminal His-tag and Thrombin sites was overexpressed in *E. coli* by IPTG induction and purified via standard procedures. The protein was used for immunization of 2 rabbits and 2 mice according to "High speed immunization protocol" (Biogenes, Germany). Rabbit anti-*Pcy*Hsp70 antibodies were used in 1:10,000 dilution, FITC-labeled goat-anti-rabbit IG was used as secondary antibody, 1:200 in PBS with 2μM DAPI. The number of EEF was determined for each well using a Leica DMI6000 inverted microscope at 400x magnification, counting approximately 20,000 cells. In later assays, the number of intracellular parasites was determined using a high-content imaging system (Operetta®, Perkin-Elmer), the analysis protocol is available as supplementary data (Suppl.protocol1). Validation of the Operetta was performed by comparing manual counting results of a number of plates with the outcome of Operetta analysis. The PVM was visualized using rabbit anti-PcyETRAMP serum. PcyETRAMP was identified from the preliminary *P. cynomolgi* genome based on orthology with PF10-0164 (ETRAMP10.3). Two rabbits were immunized with a mixture of two peptides ((C)-IISPNDELKKEGLD and (C)-IMKHRKKERKEMED), according to "High speed peptide immunization protocol" (Biogenes, Germany). For Etramp-staining of the PVM, PFA-fixed hepatocytes were partially permeabilized with 0.5% digitonin. Rabbit anti PcyETRAMP was used in 1:500 dilution, together with mouse anti-*Pcy*Hsp70 (1:10,000). As secondary antibodies TRITC-labeled anti Rabbit IgG and FITC-labeled anti-mouse IgG were used, in 1:200 dilution in PBS containing 2μM DAPI.

**Criteria for distinguishing large and small EEF parasites.** Using the limited information available about *in vivo* hypnozoite morphology, we used the following definitions ((6), (7), (8) and (9)): "Hypnozoite": intracellular parasite with 1 nucleus and small round shape, maximally 7 μm in diameter; "Developing parasite": intracellular parasite with more than 1 nucleus, larger than hypnozoite and round or irregular shape.

**Statistical analysis.** Correlations between variables were calculated using the non-parametric Spearman's Rho. $IC_{50}$ values for compounds were estimated with general least squares regression using the following equation: Percentage versus untreated = 100 * 1 / [1 + Exp(( Ln $IC_{50}$ – Ln Compound ) * Slope) ]. Where $IC_{50}$ = the concentration of compound required for 50% parasite death, Compound = the concentration of the compound, and Slope = a parameter indicating the steepness of the curve, with higher (absolute)

values indicating steeper curves. P-values smaller than 0.05 were considered significant.

**Operetta data acquisition and analysis**

**Counting *P. cynomolgi* liver stage parasites using Operetta (Perkin-Elmer), image acquisition and analysis**

**Image aqcuisition:**
DAPI (excitation 360-400, dichroic 405, emission 410-480)
Fluorescein (excitation 460-490, dichroic 495, emission 500-550)
Autofluo (excitation 460-490, dichroic 495, emission 560-630)

**Analysis Operetta:**
Input: Image (individual plane)
Find nuclei (DAPI), method C
Find Image region
    Method: common Threshold
    Channel: Fluorescein
    Threshold: 0.85
Output population: Image Region FITC
Calculate intensity properties (1)
    Channel: Fluorescein
    Population: Image Region FITC
    Region: Image Region
Output properties: Intensity Image Region FITC
Calculate intensity properties (2)
    Channel: Autofluo
    Population: Image Region FITC
    Region: Image Region
Output properties: Intensity Image Region Autofluo
Calculate properties
    Population: Image region
Method: by Formula: a/b
    a= Intensity Image Region FITC
    b= Intensity Image Region Autofluo
Output properties: Ratio FITC/AF
Calculate intensity properties (3)
    Channel: Dapi
    Population: Image Region FITC
    Region: Image Region
Output properties: Intensity Image Region Dapi
Select Population (1)
    Population: Image Region FITC
    Method:filter by property
    Filter F1: Ratio FITC/AF>5
    Filter F2: Intensity Image Region Dapi>50
    Filter F3: Intensity Image Region FITC>800
Output population: Ratio FITC/AF>5, Dapi>50, FITC>800

Calculate morphology properties:
Population: Ratio FITC/AF>5, Dapi>50, FITC>800
Region: Image Region
Method: standard
Output properties: parasite size
Select population (2)
Population: Ratio FITC/AF>5, Dapi>50, FITC>800
Method: Filter by property
Filter F1: parasite size>12
Output population: total parasites
Select population (3)
Population: Ratio FITC/AF>5, Dapi>50, FITC>800
Method: Filter by property
Filter F1: parasite size>12
Filter F2: parasite size<30
Output population: small EEF
Select population (4)
Population: Ratio FITC/AF>5, Dapi>50, FITC>800
Method: Filter by property
Filter F1: parasite size>=30
Output population: large EEF
Define results:
#nuclei
# objects FITC/AF>5, Dapi>50, FITC>800
# objects: total parasites
# objects: small EEF
parasite size (μm$^2$) [mean size of small EEF]
# objects: large EEF
parasite size (μm$^2$) [mean size of large EEF]

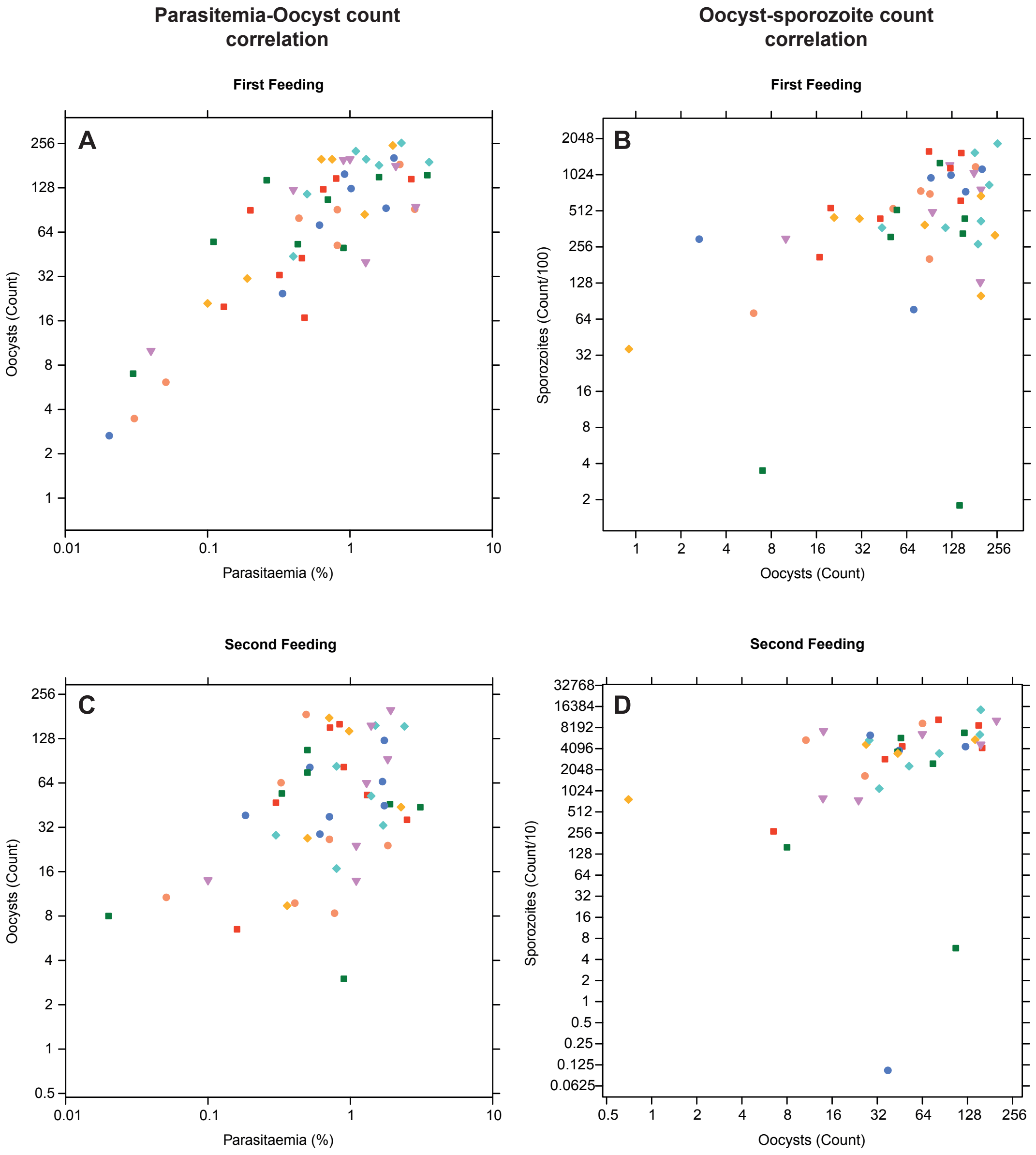


**Figure S1: Correlation plots of parasitemia vs oocyst number and oocyst number vs sporozoite yield.**

Panels A and C: Parasitemia calculations (determined from Giemsa stained thin film smears), vs oocyst numbers (mean of 10 mosquitoes) for feed 1 and 2. Panels B and D: oocyst numbers vs sporozoite numbers for feed 1 and 2. Oocyst count was positively correlated with blood stage parasitemia at the time of mosquito feeding (Spearman's Rho) 0.732, $p < 1.\ 10^{-8}$ for feed 1 and 0.372 p=0.009 for feed 2. The number of sporozoites was weakly positively correlated with the amount of oocysts (Spearman's Rho) 0.398, p = 0.0062, for feed 1 and 0.514 p=0.0017 for feed 2

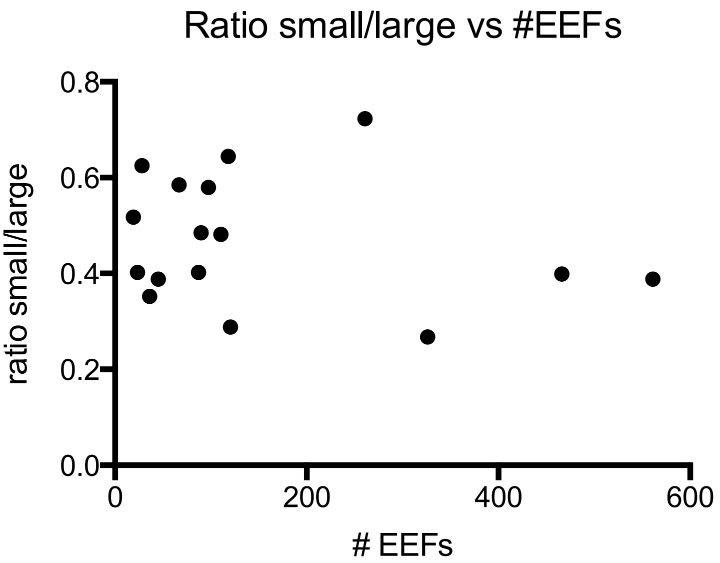


**Figure S2: Ratio of hypnozoite-forms/developing EEF vs total number of liver stage parasites over 16 independent assays.**
Liver stage parasites were counted manually (distinguishing small and large EEF by size and number of nuclei) or with the Operetta high content screening device using the script described below in the “Supplementary protocol: Counting parasites using Operetta (Perkin-Elmer), image acquisition and analysis”. The ratio small versus large parasites was calculated for each infection and is shown here relative to the total number of parasites. The ratio small/large varies between assays, but is not correlated to the total number of EEFs.

**Table S1: Transmission parameters of 53 infections.**
A rhesus macaque was infected with $10^6$ blood stage *P. cynomolgi* parasites. Parasitemia was monitored and infected blood was taken for mosquito feedings at d 11 and 12 after infection. Oocysts were counted in 10 mosquitoes, 7 or 8 days after the infected blood meal after which a second (uninfected) blood meal was given. Sporozoites were usually harvested between 6 and 8 days after the second blood meal. ND: not determined.

| Infection nr | parasitemia @feed 1 | oocyst counts feed 1 | spz yield feed 1 (spz/mosq) | parasitemia @feed 2 | oocyst counts feed 2 | spz yield feed 2 (spz/mosq) |
|---|---|---|---|---|---|---|
| 1 | 0.9 | 50 | 31000 | 0.9 | 3 | nd |
| 2 | 0.8 | 148 | 155000 | 0.3 | 47 | 44000 |
| 3 | 0.6 | 70 | 7500 | 0.7 | 37 | 0 |
| 4 | 0 | nd | nd | 0 | nd | nd |
| 5 | 2 | 248 | 32000 | 2.1 | nd | 23000 |
| 6 | 2.3 | 258 | 186000 | 1.5 | 157 | 147000 |
| 7 | 0.4 | 124 | 123000 | 1.3 | 64 | 66000 |
| 8 | 3.5 | 156 | 44000 | 1.9 | 46 | 58000 |
| 9 | 0.2 | 90 | 160000 | nd | nd | nd |
| 10 | 1 | 124 | 99000 | 1.7 | 44 | 37000 |
| 11 | 0.8 | 51 | 52000 | 0.7 | 26 | 16000 |
| 12 | 0.1 | 21 | 45000 | 0.5 | 27 | 47000 |
| 13 | 1.1 | 227 | 84000 | 0.8 | 83 | 35000 |
| 14 | 0.04 | 10 | 30000 | 0.1 | 14 | 73000 |
| 15 | 0.03 | 7 | 350 | 0.02 | 8 | 1600 |
| 16 | 0.48 | 16.8 | 21000 | 0.16 | 6.5 | 2700 |
| 17 | 1.76 | 91.2 | 94000 | 0.51 | 80 | ND |
| 18 | 2.8 | 89.8 | 69000 | 0.76 | 8.2 | ND |
| 19 | 0.63 | 200 | 10000 | 0.98 | 144 | 55000 |
| 20 | 0.4 | 43.8 | 37000 | 0.3 | 28.3 | 54000 |
| 21 | 1 | 200 | 77000 | 1.83 | 92.8 | ND |
| 22 | 0.11 | 55 | 52000 | 0.33 | 54.3 | ND |
| 23 | 0.65 | 125 | 116000 | 1.31 | 53 | 19700 |
| 24 | 0.9 | 155.4 | 72000 | 1.65 | 64.1 | ND |
| 25 | 2.2 | 180.8 | 116000 | 1.8 | 23.6 | ND |
| 26 | 0.19 | 31 | 44000 | 0.36 | 9.4 | ND |
| 27 | 1.6 | 182 | 156000 | 2.4 | 155 | 65000 |
| 28 | 2.1 | 179.8 | 106000 | 1.1 | 13.9 | 8000 |
| 29 | 1.6 | 151.3 | 33000 | 3.1 | 43.8 | 37000 |
| 30 | 0.32 | 32.7 | ND | 0.84 | 160.3 | 42000 |
| 31 | 0 | ND | ND | 0 | ND | ND |
| 32 | 0.03 | 3.4 | ND | 0.05 | 10.5 | 52000 |
| 33 | 0.0001 | 0.9 | 3600 | 0.0001 | 0.7 | 7700 |
| 34 | 1.3 | 200 | 42000 | 1.7 | 33 | 11000 |
| 35 | 2.9 | 95 | 50000 | 1.1 | 24 | 7500 |
| 36 | 0.7 | 106.5 | 128000 | | 122 | 69000 |
| 37 | 0.13 | 19.9 | 54000 | 0.9 | 82 | 106000 |
| 38 | 0.33 | 24 | ND | 0,6 | 28,2 | 61000 |
| 39 | 0.8 | 89.1 | 19800 | 0,4 | 9,6 | ND |
| 40 | 1.27 | 84.4 | 39000 | 2,27 | 43,9 | 35000 |
| 41 | 3.6 | 191.2 | 27000 | 1,4 | 52,2 | 23000 |
| 42 | 0.9 | 198 | 13000 | 1,4 | 157 | 47000 |
| 43 | 0.43 | 53 | ND | 0,5 | 75,3 | 25000 |
| 44 | 2.7 | 146.5 | 62000 | 2,5 | 36 | 29000 |
| 45 | 0.02 | 2.6 | 29000 | 0,18 | 37,8 | ND |
| 46 | 0.43 | 78 | 73000 | 0,32 | 63 | 90000 |

| | | | | | | |
|---|---|---|---|---|---|---|
| 47 | 0.75 | 200 | 68000 | 0,71 | 177 | ND |
| 48 | 0.5 | 116 | 37000 | 0,8 | 16,8 | ND |
| 49 | 1.29 | 40 | ND | 1,92 | 200 | 103000 |
| 50 | 0.26 | 144 | 179 | 0,5 | 107 | 58 |
| 51 | 0.46 | 42.6 | 44000 | 0,72 | 152 | 88000 |
| 52 | 2 | 200 | 111000 | 1,7 | 122 | 42000 |
| 53 | 0.05 | 6 | 7000 | 0,48 | 183 | ND |

**Table S2: Selected Malaria box compounds evaluated in tenfold dilution series on *P. cynomolgi* liver stages.**
Activity of compounds is schematically represented -: <10 % reduction, ±:10-20 % reduction, +: 20-70 % reduction, ++: 70-90 % reduction, +++:90-100 % reduction of parasite numbers at 10µM. The two compounds with the highest activity (GNF156 and KAI407, bold) were further analyzed in $IC_{50}$ format.

| Entry | Structure | Activity against small EEF | Activity against large EEF | *P. yoelii* Liver-stage $IC_{50}$ (mM) |
|---|---|---|---|---|
| 1 | 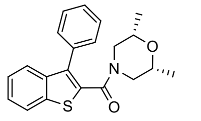 | - | - | not tested |
| 2 | 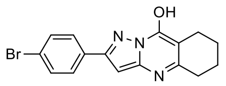 | - | - | not tested |
| 3 | 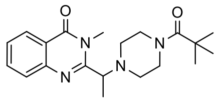 | - | ± | 0.492 |
| 4 | 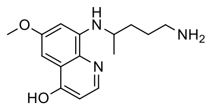 | ± | - | >10 |
| 5 (KAE609) | 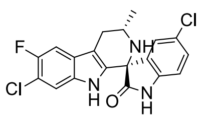 | ± | ± | >10 |
| 7 | 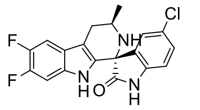 | - | + | not tested |
| 8 | 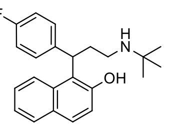 | + | ± | 5.67 |
| 9 | 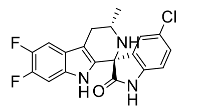 | ± | + | >10 |
| 10 | 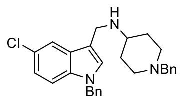 | + | + | 1.72 |

| | | | | |
|---|---|---|---|---|
| 11 | 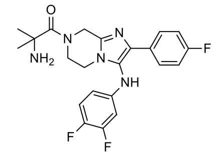 | ± | ++ | 0.277 |
| 12 (QN254) | 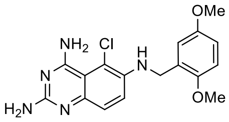 | ± | +++ | Not tested |
| 13 (KAF156) | 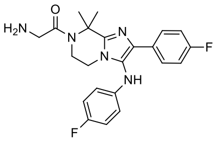 | ± | +++ | 0.001 |
| 14 (KAI407) | 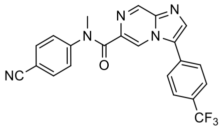 | +++ | +++ | 0.275 |

References:


1. **Ponnudurai T, Lensen AH, Van Gemert GJ, Bensink MP, Bolmer M, Meuwissen JH.** 1989. Infectivity of cultured Plasmodium falciparum gametocytes to mosquitoes. Parasitology **98 Pt 2:**165-173.
2. **Mendes C, Dias F, Figueiredo J, Mora VG, Cano J, de Sousa B, do Rosario VE, Benito A, Berzosa P, Arez AP.** 2011. Duffy Negative Antigen Is No Longer a Barrier to Plasmodium vivax - Molecular Evidences from the African West Coast (Angola and Equatorial Guinea). PLoS Negl Trop Dis **5:**e1192.
3. **Guguen-Guillouzo C, Campion JP, Brissot P, Glaise D, Launois B, Bourel M, Guillouzo A.** 1982. High yield preparation of isolated human adult hepatocytes by enzymatic perfusion of the liver. Cell Biol Int Rep **6:**625-628.
4. **Mazier D, Beaudoin RL, Mellouk S, Druilhe P, Texier B, Trosper J, Miltgen F, Landau I, Paul C, Brandicourt O, et al.** 1985. Complete development of hepatic stages of Plasmodium falciparum in vitro. Science **227:**440-442.
5. **Tsuji M, Mattei D, Nussenzweig RS, Eichinger D, Zavala F.** 1994. Demonstration of heat-shock protein 70 in the sporozoite stage of malaria parasites. Parasitol Res **80:**16-21.
6. **Krotoski WAK, D.M.; Garnham, P.C.C.; Bray, R.S.; Killick-Kendrick, R.;Draper, C.C.; Targett, G.A.T.; Guy, M.W. .** 1980. Relapses in primate malaria: discovery of two popualtions of exoerythrocytic stages. British medical journal **i:**153-154.

7. **Krotoski WA, Garnham PC, Bray RS, Krotoski DM, Killick-Kendrick R, Draper CC, Targett GA, Guy MW.** 1982. Observations on early and late post-sporozoite tissue stages in primate malaria. I. Discovery of a new latent form of Plasmodium cynomolgi (the hypnozoite), and failure to detect hepatic forms within the first 24 hours after infection. Am J Trop Med Hyg **31:**24-35.
8. **Hollingdale MR, Collins WE, Campbell CC, Schwartz AL.** 1985. In vitro culture of two populations (dividing and nondividing) of exoerythrocytic parasites of Plasmodium vivax. Am J Trop Med Hyg **34:**216-222.
9. **Dembele L, Gego A, Zeeman AM, Franetich JF, Silvie O, Rametti A, Le Grand R, Dereuddre-Bosquet N, Sauerwein R, van Gemert GJ, Vaillant JC, Thomas AW, Snounou G, Kocken CH, Mazier D.** 2011. Towards an in vitro model of Plasmodium hypnozoites suitable for drug discovery. PLoS ONE **6:**e18162.
